# Improving red-color performance, immune response and resistance to *Vibrio parahaemolyticus* on white shrimp *Penaeus vannamei* by an engineered astaxanthin yeast

**DOI:** 10.1038/s41598-023-29225-4

**Published:** 2023-02-08

**Authors:** Yu-Ju Lin, Jui-Jen Chang, Huai-Ting Huang, Chih-Ping Lee, Yeh-Fang Hu, Mao-Lun Wu, Chih-Yang Huang, Fan-Hua Nan

**Affiliations:** 1 Department of Life Sciences, National Chung Hsing University, Taichung, 40227 Taiwan, ROC; 2 Department of Medical Research, China Medical University Hospital, China Medical University, Taichung, Taiwan, ROC; 3 Graduate Institute of Integrated Medicine, China Medical University, Taichung, 40227 Taiwan, ROC; 4 Department of Aquaculture, National Taiwan Ocean University, No. 2, Pei-Ning Road, Keelung, 20224 Taiwan, ROC

**Keywords:** Immunology, Zoology

## Abstract

Astaxanthin (AST), a super antioxidant with coloring and medical properties, renders it a beneficial feed additive for shrimp. This study conducted a white shrimp feeding trial of 3S, 3’S isoform AST, which was derived from metabolic-engineered *Kluyveromyces marxianus* fermented broth (TB) and its extract (TE) compared to sources from two chemically synthetic ASTs (Carophyll Pink [CP] and Lucantin Pink [LP]), which contain 3S, 3’S, 3R, 3’S (3S, 3’R) and 3R, 3’R isoforms ratio of 1:2:1. The effects on red coloration, immune parameters and resistance to *Vibrio* infection were evaluated. Four AST sources were incorporated into the diets at concentrations of 0 (control), 100 mg kg^−1^ (TB100, TE100, CP100, and LP100), and 200 mg kg^−1^ (TB200, TE200, CP200, and LP200). Results revealed that in week 4, shrimps that received AST-supplemented feeds, especially TB100, TB200, and TE200, significantly increased redness (a*) values. Immune responses including phagocytosis activity, superoxide-anion production, phenoloxidase activity, and immune-related genes were examined on days 0, 1, 2, 4, 7, 14, 21, and 28. Generally, shrimps that received AST-supplemented feeds exhibited higher immune responses on days 7 and 14 than the control feed. Gene expression levels of superoxide dismutase and glutathione peroxidase were significantly upregulated on days 7 and 14 in shrimps that received AST-supplemented feeds, while genes of penaeidins, antilipopolysaccharide factor, and lysozyme were upregulated on days 4, 7, and 14, especially received TB200 and TE200. Furthermore, shrimps that received TB100, TE100, CP100, and LP100 7 days were then challenged with *Vibrio parahaemolyticus* and the result demonstrated higher survival rates especially TB100 at 168 h than the control feed. In conclusion, incorporating AST into the diets enhanced shrimp red coloration, immune parameters, and resistance against *V. parahaemolyticus* infection. The *K. marxianus*-derived AST exhibited higher performance than did chemical AST to be a potential feed additive in shrimp aquaculture.

## Introduction

In recent years, white shrimp (*Penaeus vannamei*) has become one of the most valuable products in aquaculture because of its high protein with low fat content and commercial value. White shrimp can store high levels of the antioxidant astaxanthin (AST), leading to red body coloration when cooked^1,2^. Consumer purchasing power is affected by color, so the price of red shrimp will be higher^3^. The demand for AST in food, feed, cosmetics, and pharmaceutical applications is increasing because of its superior antioxidative and coloring properties. Aside from the use in animal health as an antioxidant through scavenging free radicals from cellular activity or oxidative stress^4^, AST is suggested as an essential growth factor in the early development of shrimp^5^*.* Dietary astaxanthin is useful for improving the growth performance by promoting metabolic-related pathways, such as pyruvate metabolism and glycolysis/gluconeogenesis pathway^6^. Shrimp innate immunity also can be modulated by treating AST via enhancing immune responses including hematocyte count, lysozyme, phagocytic activity, and phenoloxidase, etc.^7^. AST has been commercially produced through extraction from crustaceans, chemical synthesis, and microbial synthesis. Three representative microbes—namely the alga *Haematococcus pluvialis*, the yeast *Xanthophyllomyces dendrorhous*, and the bacterium *Paracoccus carotinifaciens*—are currently used for the commercial production of two natural stereoisomers of 3S, 3’S- and 3R, 3’R-AST forms, which 3S, 3’S-AST has the best antioxidant ability^8^. However, naturally produced AST is expensive, primarily because of its low productivity and limited sources. Chemically derived AST, which has three stereoisomers (3R, 3’R), (3R, 3’S; meso), and (3S, 3’S) of an approximate 1:2:1 ratio^9^, is commonly used as an aquaculture feed additive, but its synthetic AST of 3R, 3’S (3S, 3’R) forms may be toxic to humans and the production process may cause environmental pollution^10^.

AST production in microorganisms through the use of metabolic engineering a heterologous host and other new technologies such as atmospheric and room temperature plasma (ARTP) mutagenesis to promote astaxanthin production^11^ are desirable because of the increasing demand for sustainable and affordable AST yields. A study involving the probiotic yeast *Kluveromyces marxianus* achieved cell reprogramming toward the 3S, 3’S-AST production and demonstrated that the yeast produced up to 9.97 mg/g dry cell weight (DCW) of 3S, 3’S-AST in a 5 L bioreactor^12^. The use of probiotics in shrimp aquaculture has been under investigation for several years because probiotics are believed to improve shrimp value^2^. Probiotics, as “bio-friendly microorganisms”, are chosen to replace the antimicrobial agents and they act as natural immune enhancers, which stimulate the disease resistance in shrimp farm^13^. *K. marxianus* accounts the major yeast population in kefir^14,15^. Recently, its probiotic potential has been actively explored from several studies with a focus on its health benefits and safety including its adhesion to the intestinal epithelium, antagonism toward pathogenic bacteria, anti-microbial, and anti-inflammatory functions^16–18^.

Studies have reported that diets containing chemically derived AST can increase the red coloration of shrimp and growth performance of *P. vannamei*^19–21^, *Marsupenaeus japonicu*^22^, and *P. monodon*^23^. Feeding *P. vannamei* with algae-produced or chemically derived AST can improve its immune capacity^20,24^. Chemically derived AST has been observed to protect white shrimp against *Vibrio parahaemolyticus* infection^25,26^; this infection is the most concerning problem in shrimp aquaculture worldwide because it causes acute hepatopancreatic necrosis disease^27^. However, no study has investigated whether the administration of 3S, 3’S-AST derived from probiotic yeast results in resistance against *V. parahaemolyticus* infection in cultured shrimp.

This is the first study to compare the effects of dietary *K. marxianus*-produced and chemically derived AST on immune-related genes and in the red coloration of white shrimp; the study also analyzed the potential commercial uses of *K. marxianus*-produced 3S, 3’S-AST. To investigate whether shrimp fed a diet containing AST would exhibit enhanced nonspecific immune responses, this study conducted an in vivo assay to measure phagocytic activity, O_2_^−^ production, PO activity, and immune-related gene expression. Furthermore, the study analyzed the effects of AST on resistance against *V. parahaemolyticus* infection in *P. vannamei*.

## Methods

### Sources of AST

Commercially available Carophyll Pink (CP) and Lucantin Pink (LP) with 10% AST content were purchased from DSM Nutrition (Tokyo, Japan) and BASF (Ludwigshafen, Germany), respectively. Dried powder of *K. marxianus*-produced AST fermented broth (TB) and its extract (TE) were obtained from Trade Wind Biotech Co. Ltd. (Taipei, Taiwan) and stored at 4 °C until use.

### Experimental feed preparation

The basal diet obtained from Tairoun Products Co., Ltd. A basal feed with no AST was used as a control. The experimental feed was formulated and modified on the basis of the methods described by Ngo et al.^28^ (Table 1). Specifically, the experimental feed was divided into eight groups according to the concentration of AST used to supplement the basal feed: CP100, LP100, TB100, and TE100, formulated by supplementing the basal feed with 0.1 g/kg of AST; and CP200, LP200, TB200, and TE200, formulated by supplementing the basal feed with 0.2 g/kg of AST. For experimental feed preparation, dry ingredients were mixed with 1.5% fish oil and 30% water until a stiff dough was formed. Feed pellets of approximately 2 mm were prepared. The prepared pellets were dried in a drying cabinet equipped with an air blower at 40 °C and do not exceed 50 °C, which has been test the temperature didn’t destroy the AST (data not shown), until the moisture level dropped to approximately 10% overnight. The dried experimental feed pellets were analyzed AST by HPLC and stored in a sealed plastic box at 4 °C until use.

**Table 1:** Composition and proximate contents of the experimental feeds.

Ingredients (g/Kg)	Control	Carophyll® Pink		Lucantin® pink		TE		TB	
		0.1 g/kg	0.2 g/kg	0.1 g/kg	0.2 g/kg	0.1 g/kg	0.2 g/kg	0.1 g/kg	0.2 g/kg
Fish meal	500	500	500	500	500	500	500	500	500
Shrimp meal	60	60	60	60	60	60	60	60	60
Astaxanthin	0	2	4	2	4	47.620	95.24	18.182	36.364
Yeast	50	50	50	50	50	50	50	50	50
α-starch	150	150	150	150	150	150	150	150	150
Fish oil	15	15	15	15	15	15	15	15	15
Lecithin	5	5	5	5	5	5	5	5	5
Cholesterol	5	5	5	5	5	5	5	5	5
Choline chloride	5	5	5	5	5	5	5	5	5
Vitamin premix	43	43	43	43	43	43	43	43	43
Mineral premix	40	40	40	40	40	40	40	40	40
α-Cellulose	127	125	123	125	123	79.380	31.76	108.818	90.636

### Experimental animals

White shrimp were obtained from the Aquatic Animal Center, National Taiwan Ocean University, Keelung, Taiwan. The shrimp were acclimatized to laboratory conditions in 10-ton tanks (5 m × 2 m × 1 m) and fed the commercial shrimp feed (Tairoun Products Co., Ltd.) at 5% body weight three times a day. Subsequently, the shrimp were moved indoors to tanks with a recirculating water system for the experiment. During the experiment, water temperature and salinity were maintained at 27 ± 1 °C and 33 ± 1‰, respectively.

### High-pressure liquid chromatography

Carotenoid profiles were analyzed using high-pressure liquid chromatography (HPLC) in accordance with the method described by Lin et al.^12^. 50 mg of each experimental feeds together with basal feed (Control) per batch were measured the carotenoids amount with three replicates. Total carotenoids were extracted from the experimental feeds by using DMSO in three replicates and then homogenized using a PowerLyzer 24 Homogenizer (Qiagen, Düsseldorf, Germany); the supernatant was collected after centrifugation. A Nomura Chemical Develosil C30-UG Column (Interlink Scientific Services, Dartford, UK) was used along with two buffer types, namely buffer A and buffer B: buffer A contained methanol, methyl tert-butyl ether (MTBE), and water (81:15:4 vol/vol/vol), and buffer B contained methanol, MTBE, and water (7:90:3 vol/vol/vol). The flow rate of the mobile phase was 1 mL/min, and the solvent gradient was as follows: 100% to 0% of buffer A and 0% to 100% of buffer B from 0 to 25 min, followed by 0% to 100% of buffer A and 100% to 0% of buffer B from 26 to 30 min. Samples were monitored using a Jasco 870-UV Intelligent UV/Vis Detector (JASCO International, Tokyo, Japan). AST was identified through chromatography by using commercial compound as reference standard (Sigma-Aldrich, St. Louis, MO, USA). The reference standard was prepared through serial dilution with the following concentrations to produce standard curve: 3.125, 6.25, 12.5, 25, and 50 mg/L. The standard curve was used for quantitation in combination with the extinction coefficients.

### Digital image acquisition and processing for coloration detection

A total of 180 white shrimp (9 ± 1 g) were randomly distributed into nine 149-L tanks (74 cm × 53 cm × 38 cm) for 9 groups (20 shrimp per group). The shrimp were maintained in a recirculating culture system with 50% water exchange per day. They were fed CP100, LP100, TB100, TE100, CP200, LP200, TB200, and TE200 or the control feed daily at 09:00, 15:00, and 21:00; the daily feeding rate was approximately 5% body weight. Body coloration were measured per the method of a previous study^19,29^ but with slight modifications. Five shrimp were collected from each tank on days 0, 7, 14, 21, and 28 and boiled at 100 °C for 3 min to observe their body coloration.

**Figure 1:**
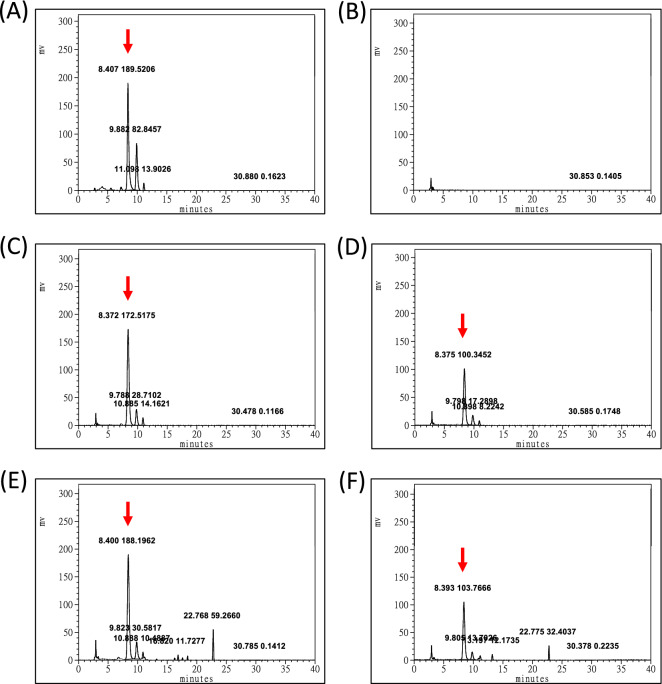
The HPLC profiles of experimental feeds. (**A**) AST reference standard, (**B**) The basal feed, (**C**) Feed with CP, (**D**) Feed with LP, (**E**) Feed with TE, (**F**) Feed with TB. Red arrow indicates the AST peak.

Shrimp body coloration was quantified using the three-dimensional CIE1976 (L*, a*, b*) color space system developed by the International Commission on Illumination, where L* represents lightness, a* represents redness, and b* represents yellowness. Adobe Photoshop 2020 software (Adobe, San Jose, CA, USA) was used for coloration index evaluation. Specifically, a captured image was first converted to the L*a*b* color space, after which gradation correction was executed to adjust the image’s white balance, as described by Wade et al.^30^. As illustrated in Fig. 1, five points were selected for color quantification; a color picking tool was used to obtain the color values at each point.

### In vivo effect of AST on immune response

A total of 320 white shrimp (9 ± 1 g) were randomly distributed into nine 205-L tanks (90 cm × 60 cm × 38 cm) for 9 groups (35 shrimp per group). The remaining 5 shrimp were used as samples on day 0. The shrimp were maintained in a recirculating culture system with 50% water exchange per day. They were fed CP100, LP100, TB100, TE100, CP200, LP200, TB200, and TE200 or the control feed daily at 09.00, 15:00, and 21:00 at a feeding rate of approximately 5% body weight. Five shrimp were sampled from each tank on days 1, 2, 4, 7, 14, 21, and 28 to analyze the immune parameters and related gene expression.

The immune parameters assay according to published methods^28^ with a slight modification. Hemolymph was withdrawn from the ventral sinus using 1-mL sterile syringes and mix with an anticoagulant (30 mM trisodium citrate, 0.34 M sodium chloride, 10 mM ethylenediaminetetraacetic acid and 0.12 M glucose, pH 7.4) in a ratio of 1:9. The withdrawn hemolymph was divided into two tubes: one of the tubes was used to conduct an assay to determine immune parameters, and the other was used to perform an assay to evaluate immune-related gene expression. The density of hemocytes for the in vivo response immune assay was quantified as the total hemocyte count (THC) and then adjusted to 1 × 10^6^ cells /mL using a hemocytometer and an inverted phase-contrast microscope (Leica DMIL, Leica Microsystems, Wetzlar, Germany). The hemolymph-anticoagulant mixture was centrifuged at 800 × g for 20 min at 4 °C, and then the supernatant was removed. Phenoloxidase (PO) activity, phagocytic rate (PR), and superoxide anion (O_2_^−^) production were measured as follows:

This study measured PO activity spectrophotometrically by recording the formation of dopachrome from L-dihydroxyphenylalanine (L-DOPA) by using the method described by Hernández-López et al.^31^, with slight modifications. Briefly, hemolymph (1 mL) was centrifuged at 800 × g at 4 °C for 20 min; subsequently, the supernatant was removed, and the cells were resuspended with 1 mL of cacodylate citrate buffer solution (0.01 M sodium cacodylate, 0.45 M sodium chloride, and 0.01 M trisodium citrate; pH 7.0). The tube was centrifuged, and the supernatant was removed; next, the pellet was resuspended with 200 μL of cacodylate buffer solution (0.01 M sodium cacodylate, 0.45 M sodium chloride, 0.01 M calcium chloride, and 0.26 M magnesium chloride; pH 7.0), and aliquots (cell suspension) were divided into two equal parts. For sample analysis, 100 μL of suspension was incubated with 50 μL of trypsin (1 mg/mL, Sigma-Aldrich) for 10 min at room temperature, which served as an activator for measuring total PO activity. For measuring background PO activity, 100 μL of cell suspension was incubated with 50 μL of cacodylate buffer for 10 min at room temperature, which served as a control. Subsequently, 50 μL of L-DOPA (3 mg/mL, Sigma-Aldrich) was added, and the solution was incubated for 15 min at room temperature. Next, 400 μL of cacodylate buffer was added to dilute the mixture. The sample was measured using a spectrophotometer (Model U-2000 Hitachi, Tokyo, Japan) at an optical density of 490 nm. PO activity was evaluated using the following equation:

$${\text{PO activity}} = {\text{OD of sample }} - {\text{ OD of background}}.$$

Phagocytic activity was measured using the method described by a previous study^28^, with slight modifications. Briefly, hemolymph (100 μL) was dropped on a cover glass and incubated at 25 °C for 60 min for adherence. After incubation, the hemolymph was washed with modified complete Hank’s balanced salt solution (MCHBSS) (10 mM calcium chloride (Panreac), 3 mM magnesium chloride (Sigma-Aldrich) and 5 mM magnesium sulfate in HBSS (Gibco)); 100 μL of latex beads (0.8 μm; 3 × 10^7^ beads/mL, Sigma-Aldrich) was then added, followed by incubation at room temperature for 60 min. Subsequently, the hemolymph was again washed with MCHBSS, fixated with methanol for 5 min, stained with Giemsa (6%) for 20 min, decolorized with distilled water, air dried, and observed under a light microscope (Olympus, Tokyo, Japan). The number of phagocytic hemocytes among 200 hemocytes was counted. Phagocytic activity was derived using the following equation:

$${\text{PR }}\left( \% \right) \, = \, \left( {\text{Phagocytic hemocytes}} \right)/\left( {\text{Total hemocytes}} \right) {\text{x 1}}00,$$

$${\text{PI }} = \, \left( {\text{Beads in phagocytic cells}} \right)/\left( {\text{Total phagocytic cells}} \right).$$

O_2_^−^ production was quantified by measuring the reduction of nitroblue tetrazolium (NBT) to formazan, as previously described^28^. Briefly, hemolymph (1 mL) was centrifuged at 800×*g* at 4 °C for 20 min, and the supernatant was removed. Zymosan (100 μL, 1 mg/mL, Sigma-Aldrich) or MCHBSS (100 μL) was added to 96-well plates for 30 min at room temperature to measure total or background O_2_^−^ production, respectively. Zymosan was added to stimulate hemocytes. The supernatant was discarded, and the hemocytes were incubated with 100 μL of NBT solution (0.3%) for 30 min at room temperature. Subsequently, the NBT solution was removed, and 100 μL of methanol was added to stop the reaction. After 5 min, the methanol was removed. The 96-well plates were washed thrice with 70% methanol (100 μL) and air dried for 30 min. The insoluble formazan crystals that were formed were dissolved with the addition of 120 μL of 2 M potassium hydroxide and 140 μL of dimethyl sulfoxide and measured using an enzyme-linked immunosorbent assay microplate reader at an optical density of 630 nm. The amount of O_2_^−^ produced was calculated using the following equation:

$${\text{O}}_{{2}}^{ - } {\text{production rate }}\left( \% \right) \, = \, \left( {{\text{OD of sample }}{-}{\text{ OD of background}}} \right)/\left( {\text{OD of background}} \right){\text{ x 1}}00.$$

### Gene expression

The method of gene expression as previously described^32^. Hemolymph was diluted with 1000 μL of anticoagulant buffer in 2-mL microcentrifuge tubes. The tubes were centrifuged at 800 ×*g* at 4 °C for 20 min. The supernatant was removed, and the hemocyte pellets were washed with 1000 mL of anticoagulant buffer. Subsequently, GENEzol reagent (Geneaid, New Taipei City, Taiwan) and chloroform (Thermo Fisher Scientific, Waltham, MA, USA) were added to each tube (5:1). The tubes were then vortexed for 15 s, incubated at room temperature for 5 min, and centrifuged at 12,000 ×*g* for 15 min at 4 °C. The colorless upper aqueous phase (0.4 mL) was transferred to a 1.5-mL tube containing equal volumes of isopropanol (Sigma-Aldrich) and incubated at room temperature for 10 min, followed by centrifugation at 12,000 ×*g* for 10 min at 4 °C. RNA pellets were washed twice with 1 mL of 75% ethanol and air dried; subsequently, they were dissolved in 13 μL of DEPC-treated water (Thermo Fisher Scientific). The quality and quantity of total RNA were measured using a SpectraMax QuickDrop Micro-Volume Spectrophotometer (Molecular Devices, San Jose, CA, USA).

First-strand synthesis was conducted using a HiScript I First Strand cDNA Synthesis Kit (Bio-Novas, Bremerton, WA, USA) in accordance with the manufacturer’s protocol. The reaction mixture (19 μL) contained DNase-treated RNA (7 μL), 2 × fast premix (10 μL), and primer oligo dT (2 μL). The cycling program was as follows: 65 °C for 5 min and 4 °C for 1 min. Subsequently, DNase-treated RNA was combined with HiScript Reverse Transcriptase and incubated at 42 °C for 30 min and then at 85 °C for 5 min; next, an inactivation step was performed at 95 °C for 1 min, and cDNA samples were further diluted 10 times in DEPC-treated water.

The primers listed in Table 2 were used to measure the expression levels of elongation factor-1α (*EF-1α*), serving as the reference gene; lysozyme (*Lyz*); penaeidin 2, 3, and 4 (*Pen2*, *Pen3*, and *Pen4*, respectively); superoxide dismutase (*SOD*); glutathione peroxidase (*GPx*); and Anti-LPS factor (*ALF*)^33^. A quantitative polymerase chain reaction (PCR) process (20 μL) involving 1 μL of cDNA, 10 μL of iQ SYBR Green Supermix with ROX (Bio-Rad Laboratories, Hercules, CA, USA), 0.4 μL each of forward and reverse primers (100 μm), and 8.2 μL of DD water was performed using a QuantStudio1 Applied Biosystems Real-Time PCR machine (Thermo Fisher Scientific). The cycles were performed at 50 °C for 2 min and at 95 °C for 10 min, followed by 40 cycles at 95 °C for 15 min and at 60 °C for 1 min. Melting curves were derived for all samples for analysis. The gene expression levels of interest were normalized to the EF-1α expression level and are expressed herein as the fold change relative to the reference expression level at each time point. The PCR process was performed in accordance with the procedure reported by Livak and Schmittgen^34^.

**Table 2:** Primers used for immune-related gene expressions.

Gene	Sequence (5’-3’)	Accession number
Superoxide dismutase (*SOD*)	F:ATCCACCACACAAAGCATCA	DQ005531
	R: AGCTCTCGTCAATGGCTTGT	
Glutathione peroxidase (*GPx*)	F: TTTTTCCGTGCAAAAAGGAC	AY973252
	R: TAATACGCGATGCCCCTAAC	
Penaeidin 2 (*Pen2*)	F: TCGTGGTCTGCCTGGTCTT	Y14925
	R: CAGGTCTGAACGGTGGTCTTC	
Penaeidin 3 (*Pen3*)	F: CACCCTTCGTGAGACCTTTG	AF390139
	R: AATATCCCTTTCCCACGTGAC	
Penaeidin 4 (*Pen4*)	F: GCCCGTTACCCAAACCATC	DQ206402
	R: CCGTATCTGAAGCAGCAAAGTC	
Lysozyme (*Lyz*)	F: GAAGCGACTACGGCAAGAAC	AF425673
	R: AACCGTGAGACCAGCACTCT	
Anti-LPS factor (*ALF*)	F: CTGTGGAGGAACGAGGAGAC	DQ208705
	R: CCACCGCTTAGCATCTTGTT	
EF1a (reference gene)	F: ATGGTTGTCAACTTTGCCCC	GU136229
	R: TTGACCTCCTTGATCACACC	

### Challenge test

A total of 198 white shrimp (9.67 ± 0.87 g) were randomly distributed into eighteen 103-L tanks (60 cm × 45 cm × 38 cm) for 6 groups in triplicate (33 shrimp per group). White shrimp were determined their resistance to *V. parahaemolyticus* infection, which pathogen was isolated from an acute hepatopancreas necrosis disease (AHPND) infected shrimp. Of these six tanks, two constituted the control groups (positive and negative groups), which were fed the control diet; the remaining four tanks constituted the experimental groups, which were fed the CP100, LP100, TB100, and TE 100 feeds (formulated by supplementing the basal feed with 0.1 g/kg of AST). These positive and experimental groups were challenged with *V. parahaemolyticus* on day 7 of the experimental period by injecting a 20-μL bacterial suspension (10^7^ CFU/mL) while the negative group were injected phosphate-buffered saline solution into the ventral sinus of the cephalothorax. The shrimp were administered the experimental feeds at a feeding rate of approximately 5% body weight at 9:00, 15:00, and 21:00 for 7 days. Mortality was recorded at 6, 12, 24, 48, 72, 96, 120, 144 and 168 h post injection.

### Data analysis

The experimental and control groups differed significantly with respect to PO activity, O_2_^−^ production, phagocytic activity, body colorization, and challenge test results. The groups were compared using one-way analysis of variance and Tukey’s honestly significant difference test in SPSS Version 25.0 (IBM, Armonk NY, USA). *P* values of < 0.05 were considered significant.

## Result

### Quantification of AST concentration in experimental feeds

To ensure that the actual AST concentrations were consistent with the theoretical AST concentrations in the feeds, HPLC was used to quantify the total AST concentration in each feed groups The HPLC profiles are showed in Fig. 1 and the AST concentration are presented in Table 3. The qualified AST peaks of each experimental feeds were appeared at the retention time around 8.4 min as the reference standard, where the basic feed wasn’t detected. The quantified results indicated that the theoretical AST concentrations were not consistent with the actual CP and LP concentrations; therefore, the concentrations of the chemically-derived AST in the prepared feeds were increased by twofold. Finally, the actual AST concentrations in CP100, CP200, LP100, LP200, TE100, TE200, TB100, and TB200 were 97.26, 219.25, 106.82, 257.84, 94.03, 192.03, 106.57, and 229.57 mg/kg feed, respectively. Moreover, the concentrations of beta-carotene in TE100, TE200, TB100, and TB200 were 16.08, 71.89, 30.67, and 97.35 mg/kg feed, respectively.

**Table 3:** Evaluation of the astaxanthin concentration of the experimental feeds.

AST sources	Groups	Theoretical AST amount	Actual AST amounts	Beta-carotene amounts
		(mg astaxanthin/kg feed)	(mg astaxanthin/kg feed)	(mg carotenoid/kg feed)
	Control	0	0	0
Carophyll pink	CP 100	200	97.26	0
	CP 200	400	219.25	0
Lucantin pink	LP 100	200	106.82	0
	LP 200	400	257.84	0
TE	TE 100	100	94.03	16.08
	TE 200	200	192.03	71.89
TB	TB 100	100	106.57	30.67
	TB 200	200	229.57	97.35

### Effects of dietary AST on shrimp body coloration

**Figure 2:**
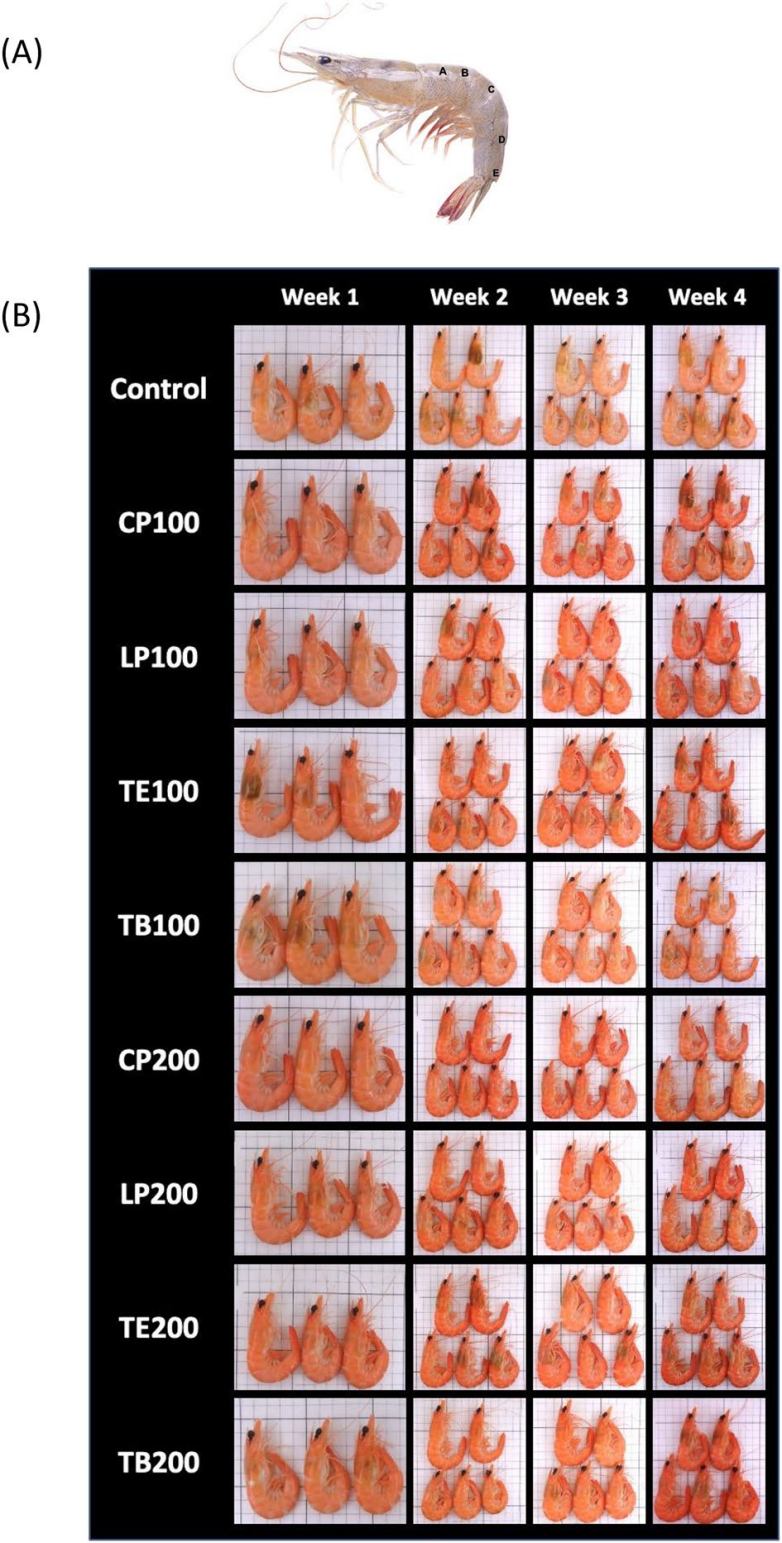
Effects of dietary AST on shrimp body colorization. (**A**) Digital analysis points of shrimp body, and (**B**) weekly photograph after boiling.

**Figure 3:**
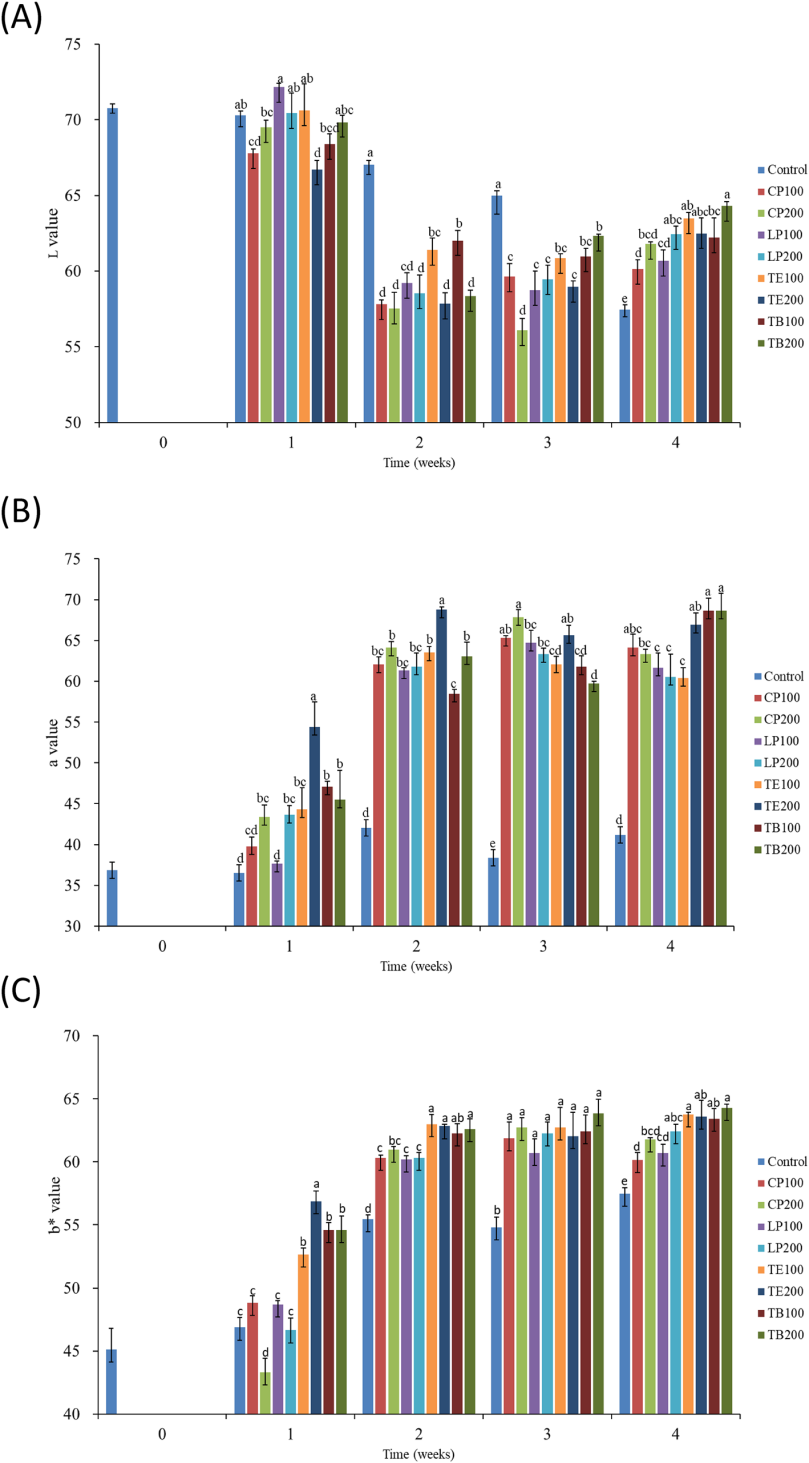
The effect of astaxanthin on white shrimp body color parameter. (**A**) L* value (lightness), (**B**) a* value (redness), and (**C**) b* value (yellowness). One-way ANOVA and Tukey’s test were performed to compare the differences between groups at indicated time point. Significant differences (*p* < 0.05) between groups are indicated by different letters above bars. The data are expressed as mean ± standard deviation (n = 3).

As mentioned, five points were selected for color quantification for each of the shrimps that received the various feed types, as shown in Fig. 2A. The body of each shrimp in each feed group was photographed in different sampling weeks, as depicted Fig. 2B. The results revealed that the L* (lightness) values derived for the shrimp that received all feed types ranged between 65 and 72 in week 1 and then decreased to 56 to 65 in the following sampling weeks (Fig. 3A, p < 0.05). However, the a* (redness) values derived for the shrimp that received all feed groups ranged between 35 and 55 in week 1 and then increased in the following sampling weeks for only the shrimp that received the AST-supplemented feeds (Fig. 3B). In weeks 1 and 2, the shrimp that received TE200 exhibited the highest a* value (*p* < 0.05). In week 4, the shrimp that received TB100 and TB200 had higher a* values (between 65 and 70) than did those that received the other feed groups (Fig. 3B). Furthermore, b* (yellowness) values derived for the shrimp that received all feed groups ranged between 45 and 56 in week 1 and then increased in the following sampling weeks (Fig. 3C). The shrimp that received TE200 exhibited the highest b* value (*p* < 0.05) in week 1. In week 4, those that received TE100 and TB200 had higher b* values (between 60 and 75) than did those that received the other feed groups, but the difference was not significant (*p* > 0.05).

### Effects of dietary AST on immune response

#### Phagocytic activity

**Figure 4:**
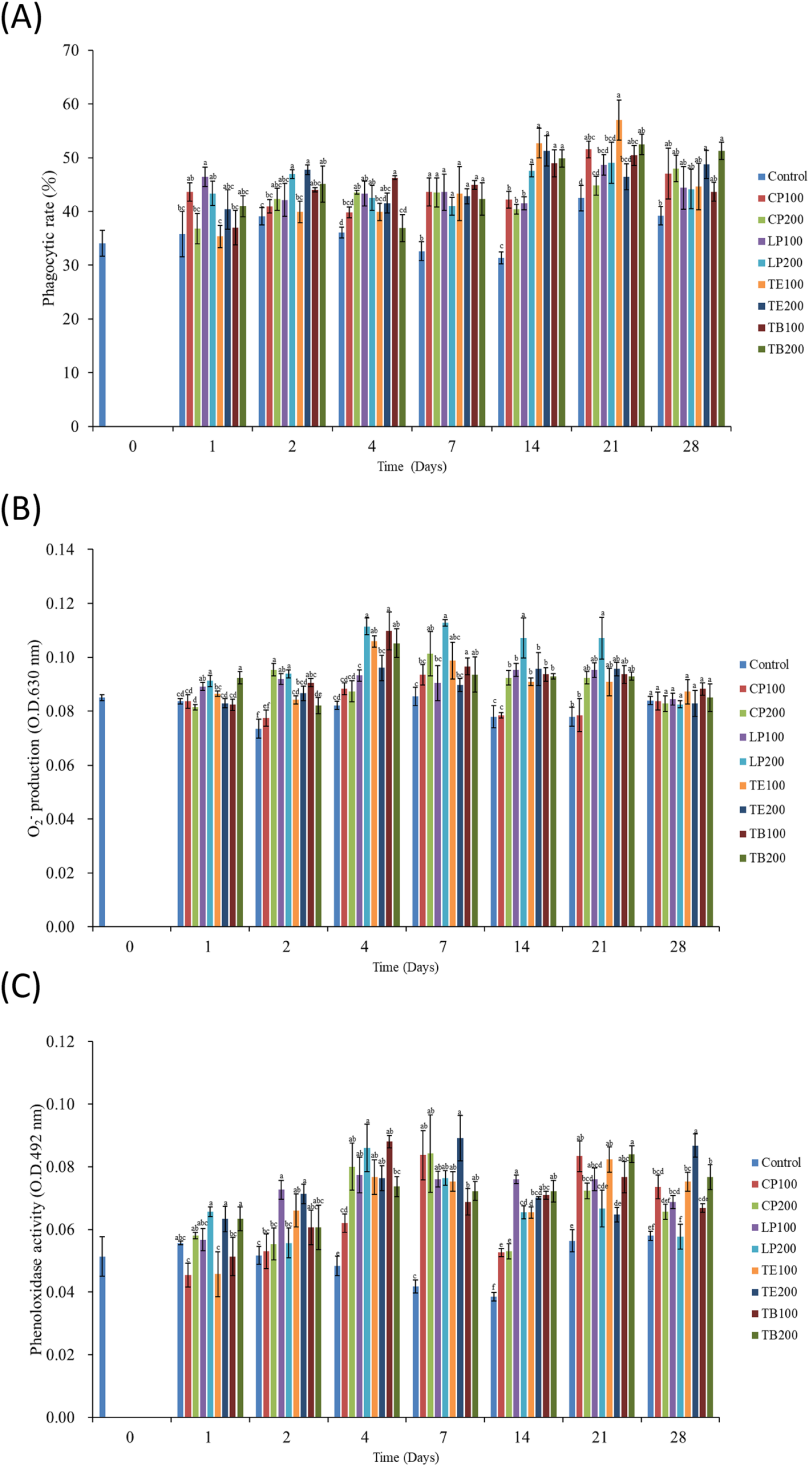
Effects of dietary AST on nonspecific immune response of white shrimp haemocyte. (**A**) Phagocytic rate, **(B**) superoxide anion production rate, and (**C**) phenoloxidase activity. One-way ANOVA and Tukey’s test were performed to compare the differences between groups at indicated time point. Significant differences (*p* < 0.05) between groups are indicated by different letters above bars. The data are expressed as mean ± standard deviation (n = 3).

From days 2 to 28, the PR values of the shrimp that received the AST-supplemented feeds were higher than that of those that received the control feed (Fig. 4A). On day 7, the PR values of the shrimp that received all AST-supplemented feeds were significantly higher than that of those that received the control feed (*p* < 0.05); the shrimp that received TB100 exhibited the highest PR. The effect of TE100 on PR activity was highest on days 14 and 21 among did those that received the other feed groups. The effect of TB200 on PR activity was highest on day 28 among did those that received the other feed groups.

#### O_2_^−^ production

From days 2 to 21, the shrimp that received all AST-supplemented feeds had higher O_2_^−^ production rates than did those that received the control feed (Fig. 4B). On day 1, the O_2_^−^ production rates in the shrimp that received LP200 and TB200 were significantly different from that in those that received the control feed (*p* < 0.05). On day 2, except for the O_2_^−^ production rate in those that received CP100, the O_2_^−^ production rates in the shrimp that received the other AST-supplemented feeds were significantly different from that in those that received the control feed (*p* < 0.05). On days 4, 7, 14, and 21, LP200 exhibited the highest effect on O_2_^−^ production (*p* < 0.05) among did those that received the other feed groups. On day 28, the results revealed no significant difference in O_2_^−^ production between the shrimp that received the AST-supplemented feeds and those that received the control feed (*p* > 0.05).

#### PO activity

From days 2 to 21, the shrimp that received the AST-supplemented feeds exhibited higher PO activity levels than did those that received the control feed (Fig. 4C). On day 2, the shrimp that received LP100, TE100, and TE200 groups exhibited significantly increased PO activity levels compared with those that received the control feed (*p* < 0.05). On days 4, 7, and 14, the shrimp that received the AST-supplemented feeds exhibited significantly higher PO activity levels than did those that received the control feed (*p* < 0.05). On day 21, the PO activity levels in the shrimp that received CP100, CP200, LP100, TE100, TB100, and TB200 increased significantly (*p* < 0.05). On day 28, the shrimp that received CP100, LP100, TE100, TE200, and TB200 demonstrated significantly enhanced PO activity levels compared with those that received the control feed (*p* < 0.05).

#### Immune-related gene expression

**Figure 5:**
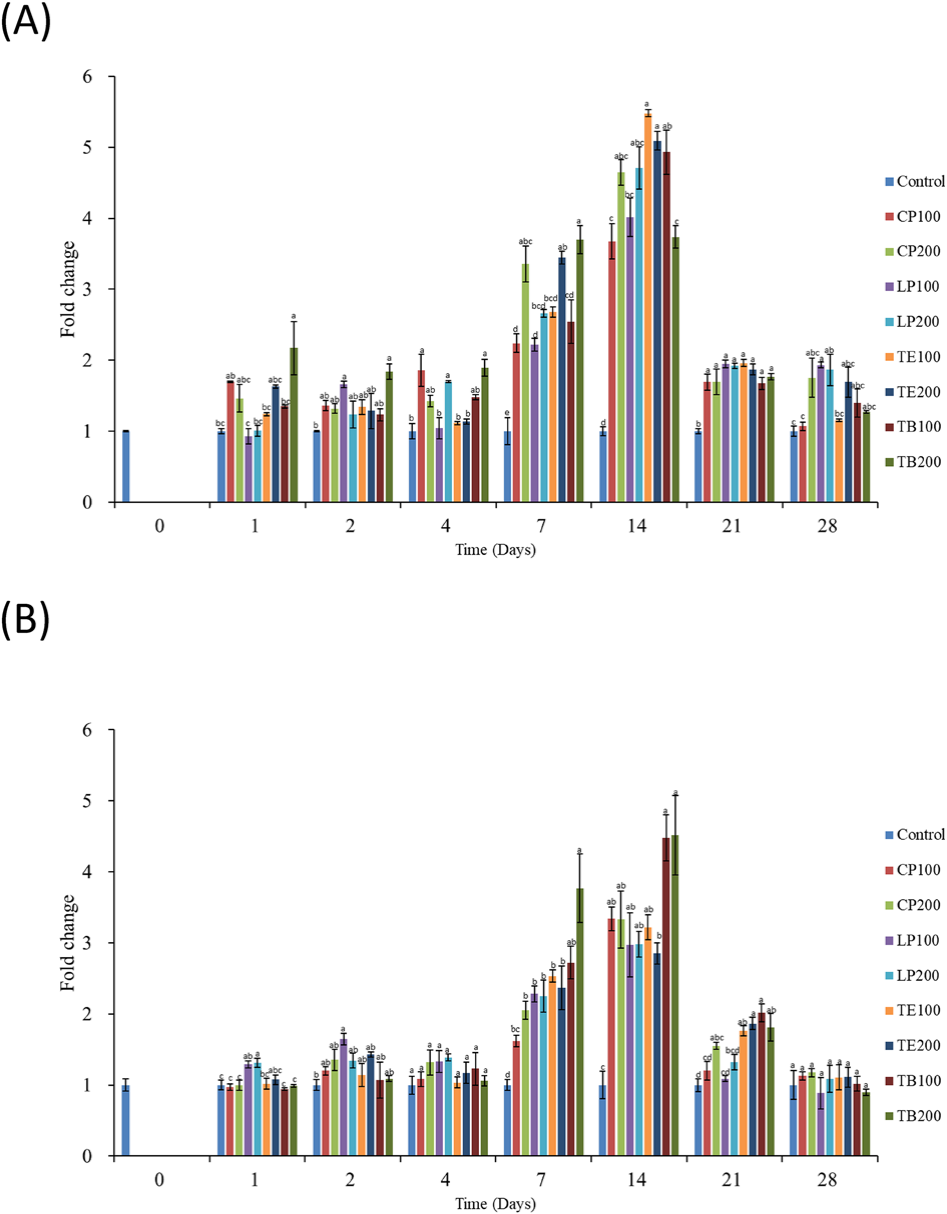
Evaluated antioxidant genes expression of haemocytes in the white shrimp fed with AST contained feed. (**A**) *SOD* and (**B**) *GPx*. One-way ANOVA and Tukey’s test were performed to compare the differences between groups at indicated time point. Significant differences (*p* < 0.05) between groups are indicated by different letters above bars. The data are expressed as mean ± standard deviation (n = 3).

This study evaluated the expression of two antioxidant genes, namely *SOD* and *GPx* (Fig. 5). The shrimp that received the AST-supplemented feeds exhibited increased *SOD* (Fig. 5A) and *GPx* (Fig. 5B) expression on day 7, and the expression level peaked on day 14. Among the shrimp that received the AST-supplemented feeds, those that received TB200 registered the highest number of days with the highest *SOD* expression levels (days 1, 2, 4, and 7) and highest *GPx* expression levels (days 7 and 14) (*p* < 0.05).

**Figure 6:**
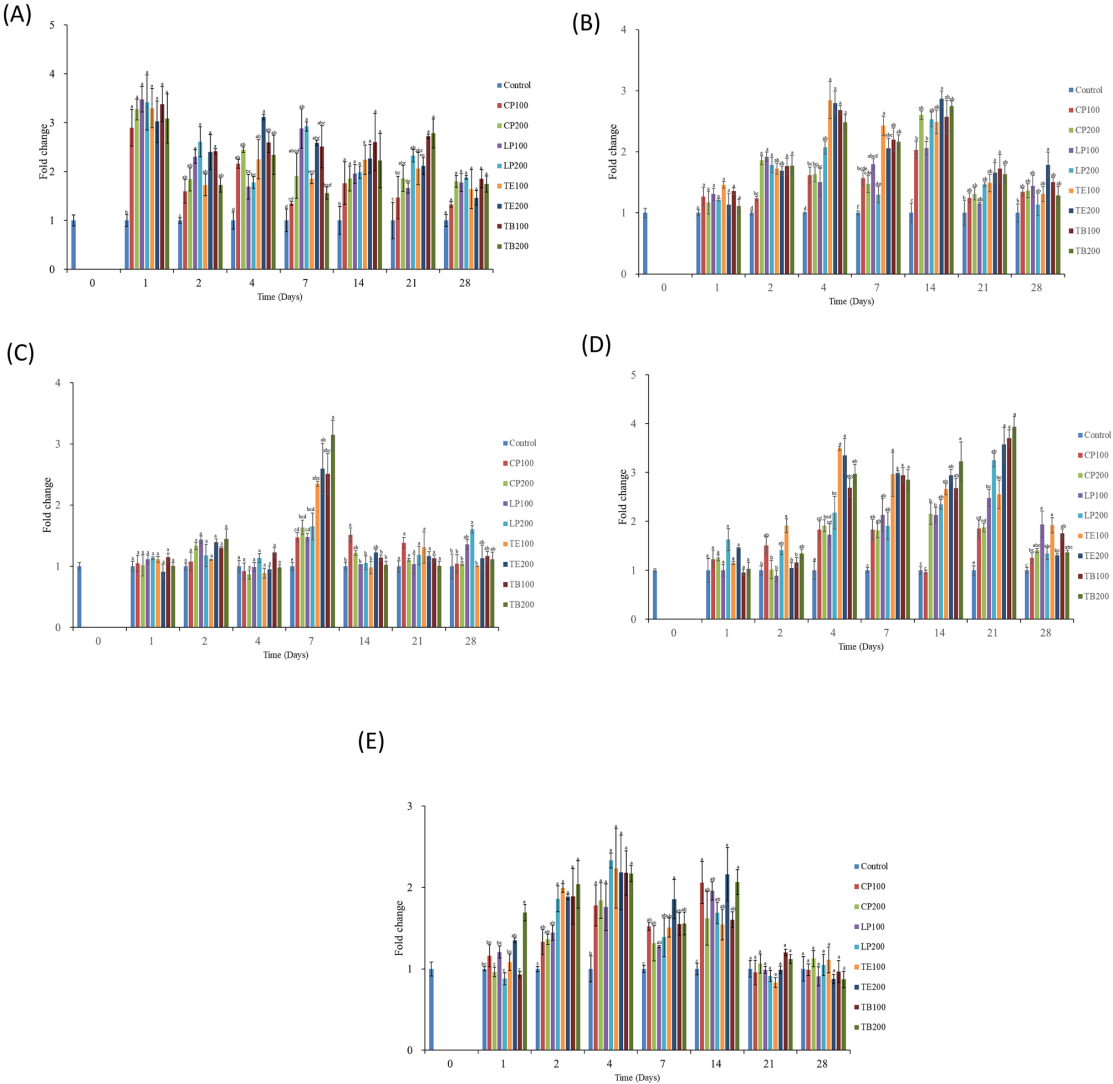
Evaluated immune-related genes expression of haemocytes in the white shrimp fed with AST contained feed. (**A**) *Pen2*, (**B**) *Pen3*, (**C**) *Pen4*, (**D**) *ALF,* and (**E**) *Lyz.* One-way ANOVA and Tukey’s test were performed to compare the differences between groups at indicated time point. Significant differences (*p* < 0.05) between groups are indicated by different letters above bars. The data are expressed as mean ± standard deviation (n = 3).

The study also evaluated the expression of five antimicrobial peptides, namely *Pen2*, *Pen3*, *Pen4*, *ALF*, and *Lyz* (Fig. 6). On days 1, 2, 4, and 14, *Pen2* was significantly upregulated in the shrimp that received the AST-supplemented feeds compared with those that received the control feed (*p* < 0.05) (Fig. 6A). Among the shrimp that received the AST-supplemented feeds, those that received TE100 and TE200 exhibited the highest number of days with upregulated *Pen2* expression (days 1 to 21) among did those that received the other feed groups. The results revealed no significant difference in peptide expression between the feed groups on day 28 (*p* > 0.05). On days 2, 4, 7, and 14, *Pen3* was upregulated in the shrimp that received the AST-supplemented feeds compared with those that received the control feed (Fig. 6B). *Pen3* expression was significantly upregulated in the shrimp that received TE100 compared with those that received the other AST-supplemented feeds on day 4 and 7 (*p* < 0.05); received TE200 compared with those that received the other AST-supplemented feeds on day 14 (*p* < 0.05). On day 7, *Pen4* was significantly upregulated in the shrimp that received the AST feeds (*p* < 0.05) (Fig. 6C). The shrimp that received TB200 had the highest *Pen4* expression level on day 7, which differed significantly from the expression levels in the shrimp that received the control feed, CP100, CP200, LP100, and LP200 (*p* < 0.05). The study revealed no significant difference in peptide expression between the various feed groups on days 1, 2, 4, and 21 (*p* > 0.05). The shrimp that received the AST-supplemented feeds exhibited the higher *ALF* expression on days 2, 4, 7, 14 (Fig. 6D). Subsequently, *ALF* expression gradually decreased until day 21 and 28 in the shrimp that received the AST feeds. Among the shrimp that received the AST-supplemented feeds, those that received TE200 exhibited the highest *ALF* expression levels on days 7 and 14, and those that received TB200 exhibited the highest *ALF* expression on days 1 and 2 (*p* < 0.05). On days 4, 7, and 21, *Lyz* expression was significantly upregulated in the shrimp that received the AST feeds compared with those that received the control feed (Fig. 6E). Among the shrimp that received the AST-supplemented feeds, those that received TE100 exhibited the highest number of days with upregulated *Lyz* expression (days 2, 4, 7, 14, 21, and 28). Overall, these results (Fig. 6) indicate that the AST feeds, especially in the TE200 and TB200 feeds, stimulated the expression of antimicrobial peptide genes.

### Survival rates of dietary AST on white shrimp after challenged with *V. parahaemolyticus*

**Figure 7:**
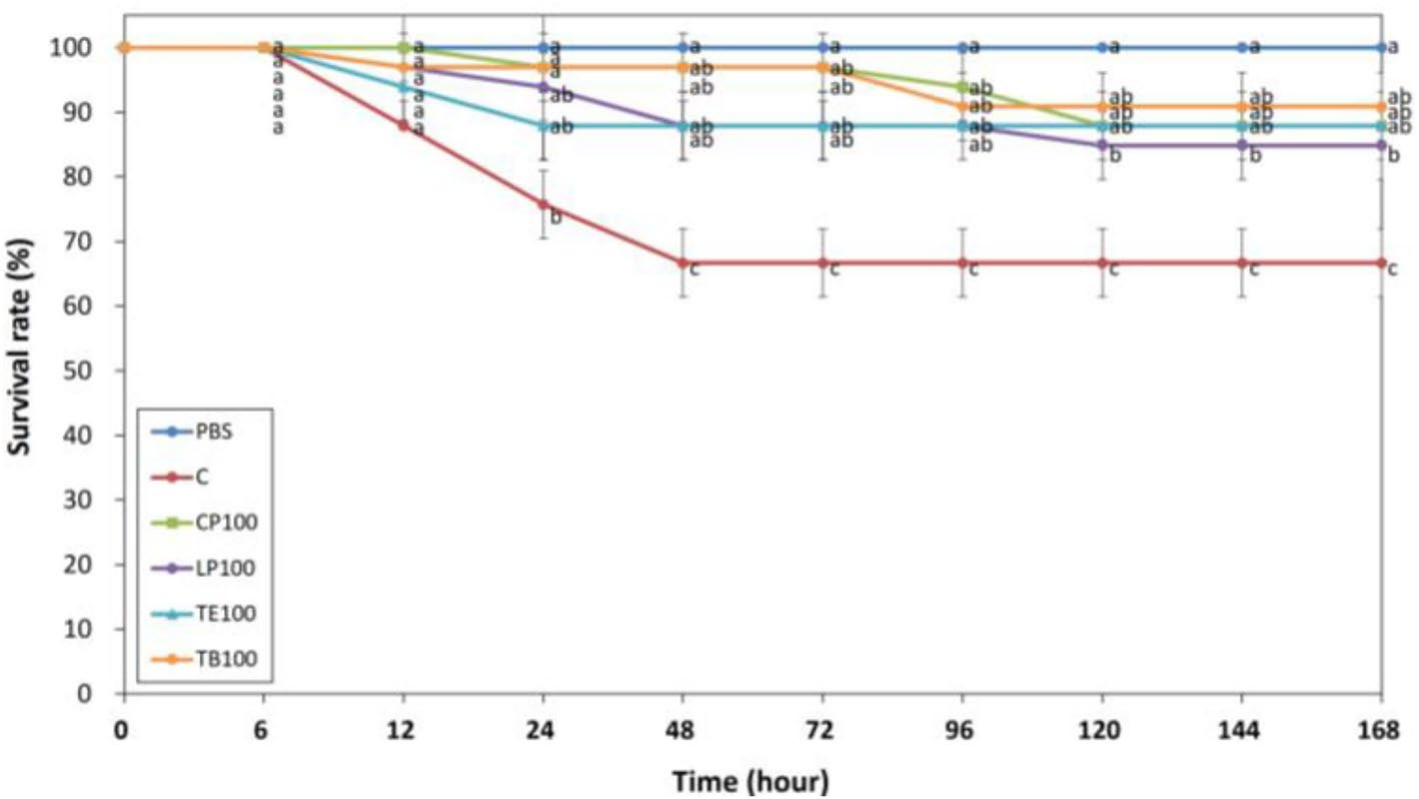
Effects of dietary AST on white shrimp resistance to *V. parahaemolyticus.* Survival rate (%) of white shrimp fed diets containing AST for 7 days after injections with *V. parahaemolyticus*. One-way ANOVA and Tukey’s test were performed to compare the differences between groups at indicated time point. Significant differences (*p* < 0.05) between groups are indicated by different letters above the time points. The data are expressed as mean ± standard deviation (n = 3).

Figure 7 presents the survival rates of the shrimp that were fed the AST-supplemented feeds for 7 days and challenged with *V. parahaemolyticus*. No mortality was observed in the negative control group (PBS) over a 168-h period. However, mortality was observed in the positive control group (C) 12 h after injection with *V. parahaemolyticus*, and the survival rate decreased considerably 12 to 48 h after injection. The shrimp that received CP100, LP100, TE100, and TB100 had significantly higher survival rates (*p* < 0.05) 48 to 168 h after injection than did those in the C group. The survival rates of the shrimp in the PBS, C, CP100, LP100, TE100, and TB100 groups were 100.00 ± 0.00%, 66.67 ± 5.25%, 87.88 ± 5.25%, 84.85 ± 5.25%, 87.88 ± 5.25%, and 90.91 ± 0.00%, respectively.

## Discussion

Color plays an important role in the acceptance and market value of shrimp^3,35^. The body surface color of shrimp is affected by food, background color, and light^35–37^. Astaxanthin can promote the health of shrimp to increase the production of shrimp, and it can also significantly increase the red color of shrimp body surface to increase the selling price^19^. *K. marxianus* derived 3S, 3’S-AST has only one isomer. Carophyll Pink has 3 isomers (3S, 3’S), (3R, 3’R) and (3R, 3’S) in the ingredients and consisted the product forms in the ratio of 1:1:2. Therefore, this study explored the different proportions of AST isomers on shrimp. Previous studies have reported that AST has a positive effect on the intermediary metabolism of aquatic animals and might improve growth by enhancing nutrient utilization^38,39^. Ju et al. and Wang et al.^19,22^ have revealed that after being cooked, shrimp fed an AST-supplemented diet had a more pronounced red coloration than did those that received a control diet (light pink coloration). Their findings are consistent with our results. Specifically, our results reveal that redness (a*) and yellowness (b*) increased significantly in week 2 in all shrimp that received the AST-supplemented feeds compared with those that received the control feed; however, the results indicate a dose-independent difference in coloration between the shrimp that received the AST-supplemented feeds. Specifically, we observed that the shrimp that received TE200, which was supplemented with *K. marxianus* derived 3S, 3’S-AST, had significantly higher redness (a*) values (*p* < 0.05) in weeks 1 and 2 compared with those that received the other AST-supplemented feeds; however, those that received TB100 and TB200, which were supplemented with fermented broth derived from *K. marxianus* derived 3S, 3’S-AST, had the highest redness (a*) value (*p* < 0.05) in week 4. The red coloration of the shrimp that received TB100 and TB200 appeared at a later time point (week 4) because *K. marxinus*-produced fermented broth, which contains yeast cells, was heated to create a dry powder; the powder must be digested first before free-form AST could be released^40,41^. By contrast, *K. marxianus* derived 3S, 3’S-AST extract could be absorbed quickly in the digestive tract, resulting in a pronounced red coloration as early as week 1. Generally, in weeks 1 to 4, the shrimp that received *K. marxianus* derived 3S, 3’S-AST (TE100, TE200, TB100, and TB200) exhibited higher yellowness (b*) values than did those that received chemically-derived AST (CP100, CP200, LP100, and LP200). This can be attributed to the presence of beta-carotene in both the fermented broth and extract forms of *K. marxianus* derived 3S, 3’S-AST. Díaz-Jiménez et al. found that an increase in the level of β-carotene in the diet can increase the accumulation of astaxanthin in the tissue^42^. They suggested that because the oxidisation of the positions 3, 3ʹ, 4, 4ʹ of β-ionone rings of β-carotene occurs^43^, allowing a gradual increase relative to the dietary concentration, which can be readily integrated into metabolic processes^44^.

Several reports have shown that immune parameters such as total hemocyte count (THC), cell viability, PR, phagocytic index (PI), PO, O_2_^−^, and antibacterial activity can be considered reliable indicators of the health status and physiological conditions of shrimp^45,46^. Wang et al.^22^ demonstrated that AST supplementation, especially with approximately 400 mg/kg dietary AST, enhances the pigmentation of juvenile kuruma shrimp, in addition to enhancing their immune response, stress resistance response, growth performance, and fatty acid content. Amar et al.^47^ also revealed that dietary carotenoids from both the marine green algae *Dunaliella salina* (beta carotene) and the red yeast *Phaffia rhodozyma* (astaxanthin) can modulate some of the innate defense mechanisms of shrimp. In the present study, hemocytes were used at several sampling time points to evaluate immune response after AST administration. Phagocytosis can be triggered by activating the components of the putative prophenoloxidase (proPO) system, where proPO is activated by PO when reacting with zymosan, lipopolysaccharide, calcium ions, and trypsin^48^. PO can adhere to the surfaces of various pathogens, such as fungi, as a sticky protein^49^ to enhance phagocytosis^50,51^, which is commonly used as an indicator of shrimp immunity. Chuchird et al. found that Pacific white shrimp fed with diets containing 50 ppm AST for a 90-day trial had PO activity significantly higher than the control^25^. Wang et al. showed that fed *L. vannamei* with 80 mg/kg astaxanthin supplemented diet for 4 weeks significantly affects the serum PO activity compared to the control^24^. Feeding *L. vannamei* with distinct AST-containing diets including 25, 50, 100, and 200 mg/kg diet for 8 weeks was associated with significant increases in the PO value^7^. The results of our in vivo assay indicated that the AST-supplemented feeds resulted in a significantly high PR on days 7 and 14 compared with the control feed (*p* < 0.05). These immune response results are similar to the observed activation of PO in the shrimp after the administration of the AST-supplemented feeds, especially in the TE and TB groups. Yowaphui et al. reported that immune parameters including THC, Phagocytosis (%), PO, and SOD activity of shrimp fed a diet containing 50 ppm β-carotene were significantly improved^52^. Previous research showed that AST was better than β-carotene either as dietary pigment or as dietary antioxidant in the commercial diet of *P. monodon*^53^, but dietary β-carotene could provide a cheaper source of carotenoid alternative^54^. Fawzy et al. studied that dietary supplementation of β-carotene could improve growth performance and survival rate, and achieve the desired coloration at the optimum β-carotene level ranged from 265.2 to 341.28 mg/kg diet for *Litopenaeus vannamei*. Besides, the combination of β-carotene and AST seemed to exert a synergistic effect^54^. Taken together, *K. marxianus* derived 3S, 3’S-AST in either TE or TB groups contains additional β-carotene benefits may refer to its efficiency in improving the overall performance.

During phagocytosis, antimicrobial substances such as O_2_^−^ are produced to eliminate bacteria. This study observed that on days 4, 7, 14, and 21, the shrimp that received the AST-supplemented feeds exhibited relatively high O_2_^−^ production rates compared with those that received the control feed. However, excessive O_2_^−^ production may lead to cell damage due to the possible oxidative stress^55^. Wu et al.^56^ reported that *SOD* activation is based on O_2_^−^ production. The *SOD* enzyme can reduce excessive O_2_^−^ production to prevent the formation of hydrogen peroxide (H_2_O_2_). If H_2_O_2_ is present, it can be degraded by *GPx* or catalase during phagocytosis. These mechanisms constitute an antioxidant self-protection system^57,58^. As a result of the respiratory burst post-phagocytic event, ROS will be released, such as superoxide anion then hydroxyl radicals will occur. Similar results were observed for *SOD* and *GPx* expression to exhibit significantly higher anti superoxide anion ability. *SOD* and *GPx* upregulation started on day 7 and peaked on day 14 in the shrimp that received the AST-supplemented feeds compared with those that received the control feed indicating the ability to scavenge superoxide anion was improved after feeding with AST supplement^24^. Eldessouki et al. also showed that the dietary AST enhanced (*P* < 0.05) the activities of serum SOD, catalase, GPx, and increased levels of total antioxidant capacity especially in the 200 mg AST/kg feed treatment in *L. vannamei*^7^. In our result, different sources of AST can increase the immune response, but only *K. marxianus* derived 3S, 3’S-AST can increase more antioxidant ability.

Regarding humoral parameters, the antibacterial activity of plasma is another indicator of health status; it can be considered an immune indicator in shrimp. Antimicrobial peptides can act against a broad range of pathogens, including gram-positive and gram-negative bacteria, yeast, fungi, parasites, enveloped viruses, and even tumor cells^59^. Penaeidins, which are unique antimicrobial peptides generated from shrimp, can be classified into three types: *Pen2*, *Pen3*, and *Pen4*. These peptides are distinguished by the N-terminal proline-rich domain and the C-terminal cysteine-rich domain^60^. *ALF* can be incorporated to inhibit infections induced by filamentous fungi and gram-positive and gram-negative bacteria; this is highly beneficial against drug-resistant vibrio species^61^. *Lyz* is efficient against gram-positive bacterial infections due to its ability to cleave the cell wall composition of β-1,4 glycosidic bonds between N-acetylmuramic acid and N-acetylglucosamine in bacteria^62,63^. In the present study, the shrimp that received the AST-supplemented feeds generally exhibited higher *Pen2, Pen3, Pen4, ALF,* and *Lys* expression levels on days 4, 7, and 14 than did those that received the control feed, demonstrating that AST supplementation can potentially prevent infections induced by fungi and gram-positive and gram-negative bacteria. Similar result was showed by Liu et al. that dietary supplementation of AST at 250 mg kg^−1^ diet for 4 weeks feeding trial significantly increased the relative expression of crustins, Pen-3α, SOD and LZM compared to the control diet^64^.

Furthermore, *K. marxianus* derived 3S, 3’S-AST exhibited higher gene expression of antibacterial peptides than chemically derived AST. In this study, we observed higher levels of immune response and immune-related gene expressions on days 7 and 14. Shrimp were fed AST at a concentration of 100 mg per feed for 7 days, which was sufficient to induce an immune response; subsequently, they were challenged with *V. parahaemolyticus*. The results reveal that 168 h after injection with *V. parahaemolyticus*, the shrimp that received TB100 had the highest survival rate. No significant difference in survival was observed between the shrimp that received the AST-supplemented feeds, but the survival rates of these shrimp differed significantly from that of those that received the control feed. These results are similar to those obtained from the assessment of in vivo antibacterial activity, especially the shrimp received *K. marxinus*-produced AST (TE100, TE200, and TB200), which consist of 3S, 3’S-AST can stimulate higher antibacterial peptides expression. Therefore, groups receiving the 3S, 3’S AST-supplemented feeds had the highest ability against to *V. parahaemolyticus* infection on white shrimp. Churchird et al. also demonstrated the effect of dietary astaxanthin on the survival of Pacific white shrimp and their resistance to *V. parahaemolyticus*^25^. Our results are consistent with those of previous studies that have reported that AST administration can enhance the survival rate of *V. parahaemolyticus*-infected shrimp^25,26^.

Our findings suggest the wide range of physiological benefits that *K. marxianus* derived 3S, 3’S-AST confers to as a potential feed supplement in aquatic animals, which these animals may be presented various improvements in survival, pigmentation, stress tolerance, disease resistance and immune-related gene expression.

## Conclusion

Both *K. marxianus* derived 3S, 3’S-AST and chemically-derived AST successfully enhanced red body coloration in white shrimp, especially those that received TE200, TB100, and TB200. *K. marxianus* derived 3S, 3’S-AST administration improved multiple immune parameters and prevented *V. parahaemolyticus* infection in the shrimp. The results of this study suggest that *K. marxianus* derived 3S, 3’S-AST has the potential to serve as an alternative aquaculture feed additive.

## Data Availability

The raw data that support the findings of this study are available from the corresponding author upon reasonable request.
